# The Effect of Safety Signs on the Monitoring of Conflict and Erroneous Response

**DOI:** 10.3389/fpsyg.2022.830929

**Published:** 2022-02-17

**Authors:** Linfeng Hu, Dingzhong Feng, Yelang Li, Jinwu Xu, Jiehui Zheng

**Affiliations:** ^1^School of Management, Zhejiang University of Technology, Hangzhou, China; ^2^College of Mechanical Engineering, Zhejiang University of Technology, Hangzhou, China; ^3^Zhejiang Hantemu Valve Co., Ltd., Lishui, China; ^4^Alibaba Business School, Hangzhou Normal University, Hangzhou, China; ^5^School of Management, Zhejiang University, Hangzhou, China

**Keywords:** safety sign, hazard, conflict, error, N2, ERN

## Abstract

The safety sign is important in our daily life and workplace to prevent potential safety issues. However, it remains undetermined whether the safety signs would influence the cognitive control ability of the people, which serves to guide the behaviors in a goal-directed manner. Therefore, this study aimed to examine the effect of safety signs on cognitive control by uncovering the behavioral performance and neural manifestations underlying the monitoring of conflict and error. The participants performed a flanker task after watching low- and high-hazard safety signs with the electroencephalogram (EEG) data recorded continually. The behavioral results indicated a classic congruency effect with higher accuracy rate and faster response time under a congruent condition compared to an incongruent condition. However, no hazard effect on behavioral performances was observed. The results of event-related potentials (ERPs) demonstrated a more negative N2 elicited by the incongruent trials and an increased (error-related negativity) ERN difference between the error and correct responses in the high-hazard condition compared to those in the low-hazard condition, implying that the monitoring of the conflict and error were both enhanced after watching the high-hazard safety signs. This study contributes to the understanding of the relationship between safety signs and cognitive control, and further expand the measurements that can be applied to assess the effectiveness of safety signs design.

## Introduction

### Safety Signs

Safety signs, as an important part of the safety management, are widely applied in our daily life and in many industries, such as transportation, manufacturing, construction, and so on. The primary roles of safety signs are to provide information, to influence behaviors, and to serve as a reminder (Laughery and Wogalter, 2014). A well-designed safety sign can provide the hazard information, lead to appropriate behaviors, and reduce potential safety issues (Laughery, 2006; Laughery and Wogalter, 2014; Chen et al., 2018; Bian et al., 2020). For example, many traffic studies find that the ergonomically designed traffic signs have a positive impact in decreasing the violations and accidents during driving (Strawderman et al., 2015; Xu et al., 2018). By contrast, a poorly designed safety sign can confuse the people, and result in many damages, such as injuries and property loss (Laughery and Wogalter, 2014; Matthews et al., 2014).

Given the importance of safety signs in our daily life and workplace, the issues about the design of safety signs and its effectiveness have been the focus of many researchers (Laughery, 2006; Laughery and Wogalter, 2014; Gao et al., 2021). Specifically, a substantial number of studies have investigated the factors consisting of design elements (e.g., size, color, and format) and non-design elements (e.g., target audience characteristics and situational factors) that are related to the effectiveness of the safety signs (Laughery and Wogalter, 2014). The achievement of effectiveness can be reflected in the accurate comprehension of the meaning expressed by the signs and performing the compliance behaviors as the signs’ guide (Laughery, 2006). The majority of previous studies adopt the interview, questionnaire, and neurophysiology approaches to measure and to examine the comprehension process of the safety signs, and to develop some indicators that can be used to assess the effectiveness of the designs. For example, Wogalter and Silver (1990) asked the participants to rate 84 signal words that are usually used in the safety signs on the several dimensions, e.g., understandability, attention-gettingness, strength, severity, and likelihood of injury, and developed a general dimension of “arousal strength,” which can measure the perceived hazard levels as conveyed by words (Wogalter and Silver, 1990). Matthews et al. (2014) conducted the interviews with 472 people about the hazard identification, recall, comprehension, and the shape of the safety signs at beaches. They found that most respondents noticed the hazard above any other information conveyed in the signs (Matthews et al., 2014). In addition to these subjective methods, the measurement of EEG is being widely applied to uncover the perception and the evaluation of safety signs and provides the objective indicators to assess the effectiveness of the safety sign comprehension (Ma et al., 2010; Parasuraman, 2011; Bian et al., 2020; Lu and Hou, 2020; Zhu et al., 2020; Hou and Yang, 2021; Hou et al., 2021. Many of these studies are also concerned about the hazard perception of the safety signs. For instance, Ma et al. (2010) demonstrated the neural mechanism underlying the evaluation of the warning words in the safety signs, and identified two stages during the comprehension, indexed by P2 and LPP, both of which were sensitive to the hazard levels conveyed by the warning words (Ma et al., 2010). Besides, Bian et al. (2020) used the questionnaire and the ERPs to compare the evaluations of three types of safety signs (prohibition, mandatory, and warning signs) and indicated the different levels of hazard perception of these safety signs, as reflected by both the self-reported results and the ERPs including P2, N2, and N4 (Bian et al., 2020). The studies mentioned above imply that a good design of safety signs, indeed, delivers necessary hazard information, which can be noticed and evaluated effectively by the people. The majority of the studies about safety signs mainly explore the psychological and neurophysiological processes, as well as the compliance behaviors that are directly in response to the safety signs (Wogalter et al., 1993; Laughery, 2006; Laughery and Wogalter, 2014). However, whether the hazard information conveyed by the safety signs could influence other cognitive functions that are essential in our daily life and work, remains undetermined. Since people are surrounded by different safety signs, they may be influenced by these signs when they are completing the tasks involving distinct cognitive functions. It is valuable to extend the understanding of the influence of safety signs on other cognitive processes that are not specific to safety signs *per se*. In the current study, we focused on the cognitive control due to its basic function to conduct various daily decisions and behaviors (Diamond, 2013; Gratton et al., 2018).

### The Conflict and Error Monitoring Underlying the Cognitive Control

The cognitive control refers to the function that guides our thoughts and actions to keep it consistent with internal intentions (Botvinick et al., 2001; Miller and Cohen, 2001; Larson et al., 2014). This goal-directed behavior requires the ability to monitor the ongoing actions and performances outcome, which serves to signal the need to change the implementation of control and to flexibly adapt our response (Ridderinkhof et al., 2004; Larson et al., 2014). The monitoring of the conflict in environment (e.g., stimuli conflict) and performance (e.g., erroneous response) are the two central aspects of this cognitive control ability in various tasks (Larson et al., 2014; Vallet et al., 2021). The falter of goal-directed behaviors is mainly attributed to the inefficient monitoring processes. For example, if people could not adequately detect the error they made, they would repeatedly make the same mistakes (Tsai et al., 2005; Duncan et al., 2011; Sozda et al., 2011).

The cognitive control is complex and encompasses a family of top-down mental processes involved in maintaining task goals (Diamond, 2013; Gratton et al., 2018). There are many tasks developed to assess the different subsets of the cognitive control ability. In the current study, we applied a flanker task to measure the interference control, one of the core functions of cognitive control, which describes the ability to resist the distractors and resolves the conflict (Mullane et al., 2009; Diamond, 2013; Gratton et al., 2018). Conflict may stem from the simultaneous activation of competing stimulus that is irrelevant to the task being conducted (Botvinick et al., 2001; Mullane et al., 2009; Larson et al., 2014). For example, in the typical flanker task, participants are instructed to judge the direction of the middle arrow (target stimulus), while simultaneously displaying other four distracting arrows (flanker stimuli) that are either pointing to the same direction (congruent condition, e.g., < < < < <) or to the opposite direction (incongruent condition, e.g., > > < > >) of the target arrow. The conflict is induced in the incongruent condition due to the simultaneous activations of the two competing stimulus properties (e.g., left vs right). Therefore, the conflict monitoring refers to the detection and processing of this competing information, which serves to trigger the recruitment of the cognitive control (Larson et al., 2014). At the behavioral level, this conflict effect would result in the high error rate and longer response time. For example, prior studies with the flanker task illustrate that the participants make more mistakes and slower response time in the incongruent condition compared to the congruent condition (Mullane et al., 2009; Larson et al., 2014). This congruency effect is attributed to the additional attention resource that is recruited to filter out the distracting information from the flanking stimuli in incongruent trials, which is the process of the interference control (Mullane et al., 2009). Error monitoring, as one central aspect of performance monitoring, refers to the detection and processing of the erroneous response in a task (Larson et al., 2014; Vallet et al., 2021). This error processing mechanism is used to signal the cognitive control to adjust the following behaviors in order to avoid further errors, which can be reflected by the slower response time in the trials following an erroneous response trial (Larson et al., 2014).

### Neural Manifestations of Conflict and Error Monitoring

Numerous studies have applied functional MRI (fMRI) and ERP methods to directly measure the monitoring processes of the stimuli conflict and the erroneous response (Larson et al., 2014; Gratton et al., 2018). As the conflict monitoring theory posits, both the detections of the conflict and the error could activate the anterior cingulate cortex (ACC) in the brain, and subsequently signal the increased need for cognitive control from the dorsolateral prefrontal cortex (DLPFC) (Botvinick et al., 2001; Yeung et al., 2004; Larson et al., 2014). At the ERP level, the N2 and error-related negativity (ERN) are suggested to have its origin in the ACC and can reflect the similar cognitive control process but at different stages (Larson et al., 2014). Specifically, the conflict monitoring process could be manifested by the activity of the N2, which refers to the negative deflection with a fronto-central scalp distribution that appears in approximately 250–350 ms after the stimulus onset (Yeung and Cohen, 2006; Folstein and Van Petten, 2008; Larson et al., 2014). Generally, N2 is associated with conflict detection and the numerous studies employ the flanker task to demonstrate that the N2 amplitude is more negative in the incongruent trials compared to the congruent trials since the high conflict degree in the incongruent condition (Iannaccone et al., 2015; Yang et al., 2019; Qi and Gao, 2020). The error monitoring process can be manifested by the ERN, which is a negative component peaking within 100 ms after the erroneous response, and mainly distributes in the fronto-central region (Gehring et al., 1993; Gehring and Fencsik, 2001; Vallet et al., 2021). Generally, an erroneous response would elicit more negative ERN amplitude compared to the correct response (Olvet and Hajcak, 2008; Larson et al., 2014).

The ability of the human to monitor the conflict and error can be modulated by several factors, which signifies in the deflections of N2 and ERN amplitudes. For example, in a flanker task, the experience of acute psychological stress could promote the alertness level and conflict detection of the people, and thus, leads to an increased N2 compared to a no stress condition (Qi and Gao, 2020). Yang et al. (2019) indicated that the defensive motivation of people could increase the conflict adaptation by exerting specific facilitatory effects on cognitive control, which was tracked by the conflict-related N2 (Yang et al., 2019). For error monitoring, Hajcak et al. (2004) indicated that participants with a high level of negative emotion exhibited the more obvious ERN amplitude in response to the errors (Hajcak et al., 2004). Another study found that higher arousal pictures preceding the flanker stimuli would increase the ERN following the error (Clayson and Larson, 2019). Furthermore, some studies also used the ΔERN (ERN amplitude difference between erroneous response and correct response) to measure the error processing (Pfabigan et al., 2013; Riesel et al., 2013; Nelson et al., 2017; Imburgio et al., 2020). For instance, Nelson et al. (2017) found that the attention bias modification training can help decrease the threat biases of people, and further reduce the neural responses of error monitoring in the flanker task, as reflected by a smaller ΔERN (Nelson et al., 2017). Taken together, the N2 and ERN are the valid indicators that can signal an abnormal or an enhanced conflict and error monitoring under different situations. However, it was neglected to explore whether the safety signs can, indeed, influence these two monitoring processes underlying the cognitive control. Besides, as many previous studies investigated the conflict and error monitoring separately, it is beneficial to examine the N2 and ERN in concert to provide a comprehensive understanding of the cognitive control (Larson et al., 2014). Therefore, the current study aimed to explore the effect of safety signs conveying the different hazard levels on people’s ability of cognitive control in a flanker task by applying the ERP method. We mainly studied the neural correlates of monitoring the conflict and erroneous response under different safety signs. Additionally, we also revealed the behavioral indices of cognitive control, specifically the interference control in the flanker task.

### The Current Study

As the previous studies suggest, a well-designed safety sign should provide the hazard information, which can be effectively evaluated by the people (Matthews et al., 2014; Chen et al., 2018; Bian et al., 2020). The perceived hazard levels of the safety signs can mobilize the psychological states, such as raising the worker’s alertness/vigilance and arousal strength (Wogalter and Silver, 1990; Hellier et al., 2000; Laughery and Wogalter, 2014; Chen et al., 2018; Ma et al., 2018). Some studies also found the associations between the hazard perception and following compliance behaviors, with high hazard leading to safer behaviors (Papastavrou and Lehto, 1996; Ma and Yuan, 2009). As the hazard information remind people about the potential threat to their health and safety, this would enhance the motivation levels, and thus, would promote the appropriate safety behaviors (Laughery, 2006; Laughery and Wogalter, 2014). In sum, the hazard perception during the evaluation of safety signs can elicit the alertness/vigilance, arousal, and motivation of the people, which have been verified to be associated with the monitoring of conflict and error (Tsai et al., 2005; Bonnefond et al., 2011; Clayson et al., 2012; Chu et al., 2019; Qi and Gao, 2020). Therefore, we inferred that the safety signs conveying different levels of hazard may have impacts on the cognitive control. To examine this assumption, the current study applied an arrow-flanker task with an ERP measurement to clarify whether the perception of the hazard information in the safety signs could impair or enhance the monitoring ability of the conflict and error by focusing on the manifestations of the two ERP components including the N2 and ERN. At the behavioral level, differences of accuracy and response time between congruent and incongruent conditions were applied to measure the interference control ability under the low- and high-hazard safety signs.

## Materials and Methods

### Participants

Twenty-four right-handed students from the Zhejiang University of Technology were recruited to attend the experiment, including 12 men and 12 women (*M* = 20.46 years, *SD* = 1.50 years). All participants reported normal, or corrected-to-normal visual acuity, and no one have any history of a neurological or mental disease. They provided informed consent and were paid for their participation. This study was approved by the internal review board of the Institute of Neuromanagement at Zhejiang University of Technology. The data from one participant was excluded because of excessive recoding of artifacts. Besides, because of the high accuracy rate and the exclusion of trials in the pre-processing of EEG, data from another three participants did not have enough trials for the analysis of the ERN. Finally, data of 23 participants were used in the analysis of behavioral performance and N2, while data of 20 participants were used in the analysis of ERN.

### Experimental Materials and Procedure

We selected forty candidate pictures of safety signs from the list of Chinese National Standard: Safety Signs and Guideline for the Use, which conveyed different levels of hazard. The pictures were processed by Photoshop to keep the same size of 300 × 300 pixel. First, in order to choose the low and high hazard groups of the signs, we recruited 107 participants to rate the hazard levels of each safety sign through a seven-point Likert scale ranging from 1 (lowest hazard) to 7 (highest hazard). Secondly, we chose the ten safety signs with the highest rating scores as the high hazard group, and another ten safety signs with the lowest rating scores as the low hazard group. The paired *t*-test showed that the mean rating of the high hazard was significantly larger than that of the low hazard group (M_Low_ = 2.49; M_High_ = 6.31; *t* = −41.91, *P* < 0.001).

As for the flanker task, we applied a modified arrow-flanker task by using the equilateral triangle as the target and flanker stimuli (Iannaccone et al., 2015). Specifically, there were five black equilateral triangles in line, and the arrowhead of the central target was pointing to either left or right side. In the congruent condition, the other four flanker stimuli on both sides of the target pointed the same arrowhead direction to the target. In the incongruent condition, the flankers had the opposite arrowhead direction from the target.

**FIGURE 1:**
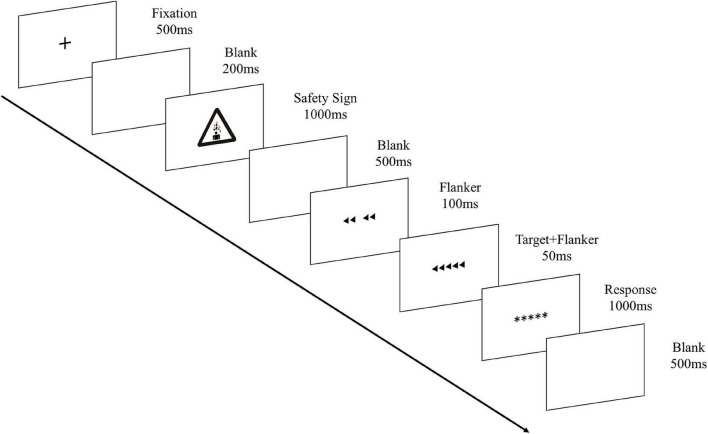
Illustration of the experiment procedure in a trial.

Figure 1 shows the experimental procedure. Each trial started with a fixation cross lasting for 500 ms, followed by a 200 ms blank. Then, the safety sign from the low and high hazard groups was randomly presented for 1,000 ms. After the sign disappeared, another blank showed for 500 ms. The flankers appeared 100 ms prior to the target, and then together with the target on the screen for another 50 ms. This design aimed to increase the distraction and task difficulty (Navarro-Cebrian et al., 2013; Iannaccone et al., 2015). Then, the target and flankers were replaced by five asterisks in the middle of the screen and the participants were instructed to make the judgment whether the middle triangle was directed left or right within 1 s by pressing the corresponding buttons on the keyboard. They were asked to respond as soon as possible. After they made the response, another blank showed for 500 ms before the next trials begun. This experiment task was ran by the E-prime software.

In total, there were 480 trials and were divided into 6 blocks with 80 trials in each. Each of the four conditions consisting of low hazard-congruent, low hazard-incongruent, high hazard-congruent, and high hazard-incongruent equally contained 120 trials. When completing a block, the accuracy rate of the current block was shown to the participants. Before the formal experiment, all the participants got practice trials to be familiar with the task. At the end of the experiment, all the participants also rated the hazard levels of the twenty safety signs with the seven-point Likert scale ranging from 1 (lowest hazard) to 7 (highest hazard). Finally, each participant was paid according to their average accuracy rate and the highest payoff was 55 RMB.

### Electroencephalogram Data Acquisition

The EEG data were continuously recorded (bandpass 0.05–100 Hz, sampling rate 1,000 Hz) by adopting the Neuroscan Synamp2 Amplifier (Scan 4.5, Neurosoft Labs, Inc., Sterling, VA, United States), with a cap of 64 Ag/AgCl electrodes according to the standard international 10–20 system. The electrode on the cephalic location served as the ground, and the left mastoid was used as the reference. Besides, vertical electrooculograms were recorded with the two electrodes placed above and below the left eye, and horizontal electrooculograms were recorded with the two electrodes placed at 1 cm from the lateral canthi of each eye. Electrode impedance was maintained below 5 kΩ throughout the whole experiment.

### Electroencephalogram Data Analysis

The raw EEG data were pre-processed offline with the Scan 4.5 (Neurosoft Labs, Inc., Sterling, VA, United States) and EEGLAB (Delorme and Makeig, 2004). The EEGs were re-referenced to the average of the left and the right mastoids and were filtered with a 30 Hz low-pass filter (24 dB/Octave). Then, the ocular artifacts were corrected. We focused on the neural mechanisms underlying the conflict monitoring after the flanker stimuli and the error monitoring after the response. Therefore, the EEG were segmented into epochs of 1,000 ms from 200 ms before the onset of the flanker stimuli to 800 ms after this onset, with the first 200 ms as a baseline, and were also segmented into epochs of 600 ms from 100 ms before the button press to 500 ms after the response with the first 100 ms as the baseline (Hajcak et al., 2005; Bode and Stahl, 2014). The epochs containing an amplifier clipping, bursts of electromyography activity, or an extreme amplitude (exceeding ± 80 μV) were excluded. Finally, the flanker-locked data were averaged separately for the four conditions consisting of stimuli congruency (congruent vs. incongruent) × hazard (low vs. high), and the response-locked data were averaged separately for the four conditions consisting of stimuli congruency (congruent vs. incongruent) × response accuracy (error vs. correct).

Two ERP components including the N2 and ERN were analyzed. It is necessary to note that since the flankers were presented preceding the target, the latency of the N2 was delayed. Specifically, the time window was 380–430 ms for N2, and the ERN was defined as the most negative peak during the 0–100 ms post-response as previous studies did (Hajcak et al., 2004; Bode and Stahl, 2014; Iannaccone et al., 2015). According to previous studies about the conflict and error monitoring, both N2 and ERN displayed a fronto-central distribution (Larson et al., 2014). Thus, we analyzed the amplitude of N2 and ERN with the pooled electrodes including F1, F2, FZ, FC1, FC2, and FCZ in the frontal-central area. We applied the within-participants repeated-measure ANOVAs on ERP data with the hazard, stimuli congruency, and response accuracy as within-participant factors.

## Results

### Behavioral Results

The manipulation of the hazard levels of safety signs was successful, with the hazard rating in high group being significantly larger than that in low group (M_low_ = 1.95, SE_low_ = 0.18; M_high_ = 5.82, SE_high_ = 0.19; *t* = −16.77, *P* < 0.001).

**FIGURE 2:**
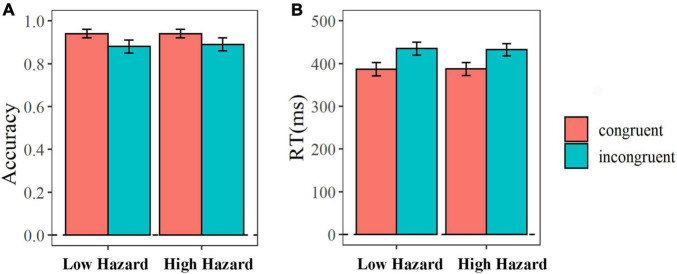
The average accuracy rate **(A)** and response time **(B)** in the flanker task. The error bar means the standard error of the mean.

We analyzed the accuracy rate and response time under different conditions (see the Figure 2). The 2 (hazard: low, high) × 2 (congruency: congruent, incongruent) repeated-measure ANOVA analysis on accuracy rate indicated that the main effect of congruency was marginally significant [*F*_(1_, _22)_ = 3.81, *P* = 0.06, η^2^ = 0.15]. The accuracy rate in incongruent condition was larger than that in congruent condition (M_congurent_ = 0.94, SE_congruent_ = 0.02; M_incongurent_ = 0.89, SE_incongruent_ = 0.03). But, the main effect of hazard [*F*_(1_, _22)_ = 0.26, *P* = 0.61, η^2^ = 0.01] and the interaction effect of congruency and hazard [*F*_(1_, _22)_ = 1.43, *P* = 0.24, η^2^ = 0.06] were not significant.

The 2 (hazard: low, high) × 2 (congruency: congruent, incongruent) repeated-measure ANOVA analysis on the response time showed that the main effect of congruency was significant [*F*_(1_, _22)_ = 19.43, *P* < 0.001, η^2^ = 0.47]. The RT in incongruent condition was longer than that in congruent condition (M_congurent_ = 386.98 ms, SE_congruent_ = 15.30; M_incongurent_ = 433.61 ms, SE_incongruent_ = 14.50). But, the main effect of hazard [*F*_(1_, _22)_ = 0.28, *P* = 0.60, η^2^ = 0.01] and the interaction effect of congruency and hazard [*F*_(1_, _22)_ = 0.63, *P* = 0.44, η^2^ = 0.03] were not significant.

### Event-Related Potential Results

**FIGURE 3:**
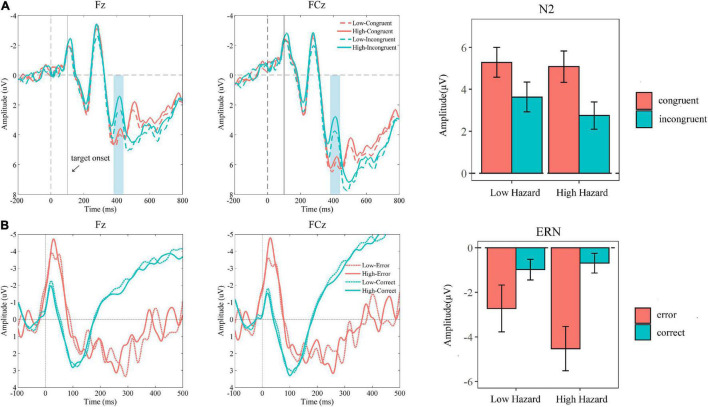
**(A)** Grand averaged ERP waveforms at Fz and FCz elicited at the stage of conflict processing with the N2 amplitude (from 380 to 430 ms) in conditions of hazard (low vs. high) × congruency (congruent vs. incongruent). **(B)** Grand averaged ERP waveforms at Fz and FCz elicited at the stage of error processing with the ERN amplitude in conditions of hazard (low vs. high) × response accuracy (error vs. correct). The error bar means the standard error of the mean.

As shown in Figure 3A, the 2 (hazard: low, high) × 2 (congruency: congruent, incongruent) repeated-measure ANOVA analysis on N2 revealed that the main effects of hazard [*F*_(1_, _22)_ = 5.77, *P* = 0.03, η^2^ = 0.21] and congruency [*F*_(1_, _22)_ = 22.69, *P* < 0.001, η2 = 0.51], as well as their interaction effect [*F*_(1_, _22)_ = 5.34, *P* = 0.03, η^2^ = 0.20], were all significant. The incongruent trials elicited a more negative N2 than that of congruent trials (M_congurent_ = 5.18, SE_congruent_ = 0.72; M_incongurent_ = 3.19, SE_incongruent_ = 0.66). Besides, the more negative N2 was elicited in the high hazard condition than that in the low hazard condition (M_low_ = 4.45, SE_low_ = 0.67; M_high_ = 3.92, SE_high_ = 0.67). The simple effect analysis showed that incongruent trials evoked more negative N2 in high hazard condition compared to low hazard condition (incongruent condition: M_low_ = 3.63, SE_low_ = 0.70; M_high_ = 2.75, SE_high_ = 0.65; *P* = 0.005), while this hazard effect was not significant for congruent trials (congruent condition: M_low_ = 5.28, SE_low_ = 0.70; M_high_ = 5.08, SE_high_ = 0.75; *P* = 0.44). The congruency effect on N2 was significant in both low and high hazard conditions (low hazard: M_congruent_ = 5.28, SE_congruent_ = 0.70; M_incongruent_ = 3.63, SE_incongruent_ = 0.70; *P* = 0.002; and high hazard: M_congruent_ = 5.08, SE_congruent_ = 0.75; M_incongruent_ = 2.75, SE_incongruent_ = 0.65; *P* < 0.001).

As shown in Figure 3B, the 2 (hazard: low, high) × 2 (response accuracy: correct, error) repeated-measure ANOVA on ERN revealed that the main effect of hazard [*F*_(1_, _19)_ = 1.80, *P* = 0.20, η^2^ = 0.09] was not significant. The main effect of accuracy [*F*_(1_, _19)_ = 12.21, *P* = 0.002, η^2^ = 0.39] and the interaction effect [*F*_(1_, _19)_ = 4.57, *P* = 0.046, η^2^ = 0.19] were significant. The error response elicited a more negative ERN than that of a correct response (M_correct_ = −0.83, SE_correct_ = 0.42; M_error_ = −3.63, SE_error_ = 0.88). The simple effect analysis showed that although the response accuracy effect was significant both in low and in high hazard conditions, it was more significant in the high hazard condition (high hazard: M_correct_ = −0.68, SE_correct_ = 0.44; M_error_ = −4.53, SE_error_ = 0.99; *P* = 0.002) compared to the low hazard condition (low hazard: M_correct_ = −0.98, SE_correct_ = 0.46; M_error_ = −2.72, SE_error_ = 1.05; *P* = 0.045). The hazard effect was not significant for error and correct responses, but it revealed the trend that the error response elicited more negative ERN in a high hazard condition than in a low hazard condition (error: M_low_ = −2.72, SE_low_ = 1.05; M_high_ = −4.53, SE_high_ = 0.99; *P* = 0.09). Furthermore, we also analyzed the ΔERN (ERN of error—ERN of correct), which was used in previous studies, as an indicator to reflect the ability of error monitoring (Pfabigan et al., 2013; Riesel et al., 2013; Nelson et al., 2017; Imburgio et al., 2020). The paired *t*-test of the ΔERN indicated that the ΔERN in a high hazard condition was significantly larger than that in a low hazard condition (ΔERN_low_ = −1.74; ΔERN_high_ = −3.84; *t* = 2.14, *P* = 0.046).

## Discussion

This study aimed to investigate the effect of safety signs with different levels of hazard on the neural correlates of monitoring the environment conflict and performance, which are the two mechanisms to signal the need to implement the cognitive control. The participants performed an arrow-flanker task after watching the high-hazard safety signs or the low-hazard safety signs with the EEG recorded continually. At the behavioral level, the classic congruency effects on accuracy rate and response time were observed. In detail, more erroneous responses and longer response time were found in the incongruent condition compared to those in the congruent condition. However, we did not find any effect of hazard level on the behavioral performance. At the neural level, the conflict and error monitoring were modulated by the safety signs. Specifically, N2 was more negative when processing the incongruent stimuli in the high hazard condition compared to that in low hazard condition. However, this difference in N2 was not obvious for the congruent stimuli. Also, an enhanced ERN amplitude difference between erroneous response and correct response was found in the high hazard condition compared to the low hazard condition. These results indicate that the hazard information of the safety signs, indeed, promote the conflict and the error monitoring underlying the cognitive control in the flanker task, but do not influence the behavioral performance of interference control. This study extended the knowledge about the influence of the safety signs on more cognitive functions by directly revealing the relationships between the hazard perception and both the conflict and error monitoring underlying the cognitive control in the flanker task. According to the conflict monitoring theory, stimuli conflict and error monitoring are the two core processes used to implement the cognitive control in a task (Botvinick et al., 2001; Yeung et al., 2004; Larson et al., 2014). Although both the N2 and ERN are suggested to be generated from the ACC, they represent separable processes and are susceptible to different factors, and even exhibit different reactions to the same factor (Larson et al., 2014). For example, Tsai et al. (2005) demonstrated that error monitoring was impaired by a sleep deprivation, reflected by the decreased amplitude of ERN, but the conflict monitoring reflected by the N2 was not attenuated by sleep deprivation (Tsai et al., 2005). Therefore, it is beneficial to integrate the manifestations of N2 and ERN to understand the general performance of the cognitive control when considering the effects of influential factors (Larson et al., 2014). Compared to previous studies that only demonstrated the effect of alertness on one of these two aspects of cognitive control (e.g., Clayson and Larson, 2019; Qi and Gao, 2020), the findings of our study indicated that the alertness induced by the safety signs had a consistent influence on the conflict and error monitoring, and provided a more comprehensive view on the monitoring process underlying the cognitive control.

### The Effect of Safety Signs on the Conflict Monitoring

In line with previous studies, our results of the congruency effect on the accuracy rate and RTs indicated the interference effect induced by the conflict in directions between the target and flankers (Mullane et al., 2009; Larson et al., 2014). In the incongruent condition, the participants needed to devote more effort to resist the distractors, resulting in slower response and high error rate (Mullane et al., 2009; Larson et al., 2014). In parallel with the behavior results, we also observed the significant congruency effect on the N2 in both low and high hazard conditions, which was consistent with the previous studies, indicating that the ongoing conflict detection process happened when performing the flanker task (Folstein and Van Petten, 2008; Larson et al., 2014). The N2 is suggested to signal the dynamic recruitment of cognitive control in different situations (Folstein and Van Petten, 2008). According to the conflict monitoring theory, the more negative N2 in the incongruent condition than congruent condition indicated that more cognitive control was recruited to resist the interference of flanker stimuli (Yeung and Cohen, 2006; Danielmeier et al., 2009; Larson et al., 2014).

Besides, the incongruent trials elicited more negative N2 amplitude in the high hazard condition compared to that in the low hazard condition, suggesting the hazard delivered by the safety signs could promote the conflict monitoring. Hazard information is necessary for an effective safety sign design (Laughery and Wogalter, 2014). Previous studies have found this information can be perceived by the people both in explicit and in implicit ways, with high-hazard safety signs eliciting stronger neural responses than low-hazard safety signs, indicating a higher alertness and arousal level (Ma et al., 2010; Bian et al., 2020). The self-report and other psychology experiments also discover an increased alertness after watching the safety signs with high hazard information (Wogalter and Silver, 1990; Hellier et al., 2000; Laughery and Wogalter, 2014; Chen et al., 2018). Several ERP studies have applied various tasks, such as Flanker task, Stroop task, and have verified that the increasing alertness levels could promote the conflict detection, with the manifestation of more negative N2 in the incongruent condition (Clayson et al., 2012; Chu et al., 2019; Qi and Gao, 2020). The hazard effect on N2 in our study was consistent with these studies, and also provided neural evidence to the attention network theory, which posits that the alerting network in our brain can influence the executive network (Mullane et al., 2009; Asanowicz and Marzecová, 2017). Specifically, various behavioral experiments using the attention network test and its variants yield the common finding that an alerting cue (visual or auditory forms) preceding the stimuli can influence the selective attention processing underlying the cognitive control in the flanker task, resulting in a stronger congruency effect compared to that in the condition without any cues (Weinbach and Henik, 2012; Asanowicz and Marzecová, 2017; Schneider, 2019). In other words, people are distracted more by the flanker stimuli in an alerting state (Weinbach and Henik, 2012; Asanowicz and Marzecová, 2017; Schneider, 2019); this is because alertness induces a global processing bias, which increases the accessibility to more spatial information in the visual field (Weinbach and Henik, 2011, 2012). Such mechanism could be supported by the N2 deflection in our study, since numerous studies indicate that a larger N2 is elicited when people attend more to the task-irrelevant (flanker) information (Yeung and Cohen, 2006; Danielmeier et al., 2009; Larson et al., 2014). In conclusion, the high-hazard information conveyed in the safety signals would induce a higher alerting state of the people, which possibly expands their focus of attention and make them evaluate the flanker stimuli more, i.e., promoting the conflict monitoring. Finally, it leads to a larger conflict level and elicits a more negative N2 amplitude.

### The Effect of Safety Signs on Behavioral Performance of Cognitive Control

Contrary to some studies that found the alerting state induced by a cue could enlarge the congruency effect at a behavioral level (e.g., Weinbach and Henik, 2012; Schneider, 2019), no hazard effect on accuracy rate and RTs was observed in our study, and the participants exhibited similar behavioral performances under low and high hazard conditions. We considered two possible explanations for these different patterns. Firstly, most previous studies indicated that the congruency effect on behaviors was larger in the condition with the alerting cue compared to that without any cue (e.g., Weinbach and Henik, 2012; Schneider, 2019), while the current study compared the behaviors in the low hazard (low alerting state) with that in the high hazard (high alerting state). In other words, the low hazard information could induce a certain level of alertness, which was higher than that in no-information condition. There might be more obvious difference of the effects on cognitive control between none and other alertness levels. This speculation was preliminarily supported by a previous study finding that there were significant differences of congruency effects between conditions with and without the alerting cue, but non-significant difference between conditions with single alerting cue and double alerting cue (stronger alerting stimulation) (Asanowicz and Marzecová, 2017). But this assumption still needs further investigation.

Second, another possible explanation might be the influence of the task difficulty. Previous studies suggested that the interactions between different attention networks might depend on the task difficulty (Roca et al., 2011; Asanowicz and Marzecová, 2017). In comparison to results from the standard attention network test, other studies have applied different versions of this task and have discovered inconsistent results. The alerting effect on the congruency effect could be non-significant (e.g., Roca et al., 2011, 2013) or just obvious in RT or accuracy (e.g., Asanowicz et al., 2012; Asanowicz and Marzecová, 2017). Actually, the results about the interactions between alerting and the cognitive control still are not entirely consistent across studies (Asanowicz and Marzecová, 2017; Schneider, 2019). In current study, the flanker task might be less challenging for the participants, and they could keep a high level of behavioral performance. A prior study using the arrow flanker task found that even in the high working memory load condition, the participants could still keep the similar RT and accuracy rate for the incongruent trials compared to that in the low load condition (Wei and Zhou, 2020). Besides, the most effective safety signs are designed less complexly that require less attention resources in order to promote the comprehension rate (Siswandari and Xiong, 2015; Tejero et al., 2018; Babić et al., 2020). Even in an implicit evaluation of the safety signs, people can also effectively distinguish the different levels of the hazard (Bian et al., 2020). Well-designed safety signs can enable the effective performance in multi-tasking (Chen et al., 2018). Therefore, the evaluation of different safety signs may not have recruited too much cognitive resources in our study, which would ensure ample cognitive resources for participants to complete the following flanker task. There were dynamic adjustments of the attention resource between different processes underlying the cognitive control in order to achieve the task goals (Otten and Jonas, 2013; Gratton et al., 2018). This mechanism could account for the inconsistent effects of hazard on behavioral performances and the N2. Although the hazard information could enhance the conflict monitoring reflected by larger N2, it did not mean that the interference control in the flanker task should be enhanced or impaired. The association between N2 and cognitive control performance is still debated. For example, Chu et al. (2019) indicated that the larger N2 amplitude was accompanied by the higher accuracy in the Stroop task (Chu et al., 2019), while Lamm et al. (2006) found the contrary result that a greater N2 was associated with a worse performance in the Stroop task (Lamm et al., 2006). Overbye et al. (2021) found no relationship between the N2 and the performance in the flanker task (Overbye et al., 2021). The implementation of cognitive control is effortful and involves several subprocesses (Miyake et al., 2000; Miyake and Friedman, 2012; Gratton et al., 2018). As the conflict monitoring theory suggests, the successful cognitive control needs the dynamic interaction between ACC and DLPFC (Botvinick et al., 2001, 2004; Yeung et al., 2004). Therefore, the manifestation of N2 just reflects one aspect (i.e., conflict monitoring) of the cognitive control process, and the final behavioral performance could be the result of the combination of more subprocesses and could be modulated by several factors, such as task types, task demand, and individual difference (Gratton et al., 2018). Since the task was easier in our study, even if the participants devoted more to monitor the conflict in the high hazard condition, ample attention resources were left for the next implementation of an interference control. Some previous studies also found the experimental conditions have elicited the difference in N2, but not in the behavioral performance. For example, Otten and Jonas (2013) found the that the social exclusion people exhibited a larger N2 than the no exclusion people, indicating the promotion of the conflict monitoring, but the response performance between these two kinds of people did not differ in the Go/No-Go task. They attributed it to the balance of cognitive control resources between the conflict monitoring and the following response inhibition (Otten and Jonas, 2013). The second explanation should also be examined in further works by applying more difficult tasks. This study provided a suggestion that future studies should carefully consider more degrees of the strengths of alertness induced by different safety signs and the difficulty of the tasks, to deepen the understanding of the interactions between safety signs and cognitive control both at behavioral and neural levels.

### The Effect of Safety Signs on the Error Monitoring

In agreement with previous studies, our study also found a larger ERN that was elicited after an erroneous response compared to a correct response in low- and high-hazard conditions, indicating the error detection process during the flanker task (Olvet and Hajcak, 2008; Simons, 2010; Larson et al., 2014). Besides, we discovered the bigger ERN difference between erroneous and correct responses in the high hazard condition compared to that in the low hazard condition, which could also be explained by the increasing alertness level induced by safety signs with the high hazard information. It is suggested by previous studies that the error monitoring is enhanced when the participants were in a high state of alertness/vigilance or arousal (Hajcak et al., 2004; Tsai et al., 2005; Bonnefond et al., 2011; Saunders et al., 2016; Andreu et al., 2017; Clayson and Larson, 2019); it is because the emotionally salient context (e.g., negative affective) would increase the attention and enhance the error monitoring (Larson et al., 2014; Clayson and Larson, 2019). In addition, our results were also in line with the motivational significance theory accounting for the effect of the error saliency on the ERN (Olvet and Hajcak, 2008; Larson et al., 2014). Numerous studies suggest that the deflection of ERN is sensitive to the motivational value of the performance outcomes. For example, a larger ERN was elicited in the situation that the error or accuracy was more emphasized (Gehring et al., 1993; Hughes and Yeung, 2011), because this made the errors more important to the people. Other studies have directly associated the error with the performance incentives, and found that more negative ERN was elicited by the error that resulted in a larger financial loss (Hajcak et al., 2005; Moser et al., 2005; Chiu, 2007; Hajcak and Foti, 2008; Riesel et al., 2012). Moreover, another study found experiencing feelings of helplessness increased the subjective error significance perception and thus elicited a larger ΔERN in a flanker task (Pfabigan et al., 2013). The ΔERN is suggested as a valid indicator across the tasks to measure the ability of the performance monitoring and individual difference (Riesel et al., 2013; Imburgio et al., 2020). For example, some studies found that the training of disengaging attention away from the threat-relevant information would reduce the ΔERN compared to the no training group, indicating the reduced error monitoring (Nelson et al., 2015, 2017). Besides, another study discovered that compared to healthy adults, the multi-problem young adults exhibited a worse performance and impaired error processing, as reflected by a smaller ΔERN (Zijlmans et al., 2019). As prior studies, the important role of the safety signs is to deliver the hazard information and a reminder to the audiences to avoid the potential risk and accidents that are harmful for themselves (Laughery, 2006). Therefore, we thought that the hazard information of safety signs might not only induce the high arousal and alertness level of the people, but also add more motivational significance on the error, thus, resulting in a bigger ΔERN in a high hazard condition than that in a low hazard condition. In sum, both higher alertness and high motivational significance induced by the safety signs with high hazard led to an enhanced error monitoring.

## Conclusion

This study applied the event-related potential method to investigate the effect of safety signs on the cognitive control of the people by taking into account the monitoring ability of conflict and error. The results illustrated that both the conflict monitoring and the error monitoring, after watching the high hazard safety signs, were enhanced, which were characterized by the larger N2 and ΔERN. It could be attributed to the increased alertness levels induced by high-hazard safety signs that expanded the attention to the distracting information in the conflict processing, and the increased attention as well as the motivational significance in the error processing. This study also demonstrated that the high-hazard safety signs might promote the monitoring ability without impairing the behavioral performance of the cognitive control in the flanker task. Such mechanism is useful in the daily life and work. Specifically, the safety signs can increase the attention to the surrounding events that is helpful in dealing with the threats more efficiently. Besides, the enhanced sensitivity to erroneous response can help to implement more effective and adaptive behaviors to achieve the goal. To our knowledge, this study initially examined the effectiveness of safety signs by uncovering its effect on the following cognitive control, which has the practical values to supplement the measurements in assessing the designs of safety signs. In addition to measuring the accurate comprehension of the safety signs and the compliance behaviors, it is also feasible to apply various psychology tasks and explore the effects of safety signs on more cognitive functions involved in the safety behaviors and decisions, such as the driving.

Although this study provided primary insights regarding the influence of the safety signs on the cognitive control, there were still several limitations that need to be remedied in the future work. Firstly, this study mainly focused on the effect of safety signs on the cognitive control that is implemented on the current trials rather than the influence on the trial-by-trial adjustment. It was limited by the current experiment design. Future work can investigate these issues, such as the conflict adaptation and post-error slowing under the safety signs with different hazard levels. Secondly, this study only revealed the effect of safety signs on the interference control in the flanker task, which might limit the extension of the conclusions to other conflict-related paradigms, such as Stroop task and Simon task, as well as other aspects of cognitive control; for example, the response inhibition in Go/No-Go task or stop-signal task. In the future work, more complex tasks can be conducted by considering more factors, such as task type, task difficulty, and individual difference. Third, the safety signs used in the current study consisted of different types, thus, the pertinence is somewhat weak. Future work can explore the effect of a specific type of safety signs used in a certain industry.

## Data Availability Statement

The raw data supporting the conclusions of this article will be made available by the authors, without undue reservation.

## Ethics Statement

This study was reviewed and approved by the internal review board of the Institute of Neuromanagement at the Zhejiang University of Technology. The participants provided their written informed consent to participate in this study.

## Author Contributions

LH: research conception, experiment design, data collection, data analysis, and writing the initial and final draft. DF: research conception, experimental design. YL: research conception, and experiment stimuli preparation, and experiment program preparation. JX: experiment stimuli preparation, and experiment program preparation. JZ: research conception, experiment design, data analysis, and review and writing the final draft. All authors contributed to the article and approved the submitted version.

## Conflict of Interest

LH, YL, and JX were employed by Zhejiang Hantemu Valve Co., Ltd. The remaining authors declare that the research was conducted in the absence of any commercial or financial relationships that could be construed as a potential conflict of interest.

## Publisher’s Note

All claims expressed in this article are solely those of the authors and do not necessarily represent those of their affiliated organizations, or those of the publisher, the editors and the reviewers. Any product that may be evaluated in this article, or claim that may be made by its manufacturer, is not guaranteed or endorsed by the publisher.
